# Case Report: Leiomyosarcoma of the right external iliac artery: a diagnostic-based study on a rare case

**DOI:** 10.3389/fonc.2023.1274694

**Published:** 2023-12-11

**Authors:** Mpano Olivier, Ahmed Abu Ryash, Weiwei Yin, YuHui Bao, Qiangyong Zhou, Xi Wang, Guorong Chen, Xiaojian Yan

**Affiliations:** ^1^ Wenzhou Medical University, Gynecology Department of the First Affiliated Hospital of Wenzhou Medical University, Wenzhou, Zhejiang, China; ^2^ Wenzhou Medical University, Department of Pathology, First Affiliated Hospital of Wenzhou Medical University, Wenzhou, Zhejiang, China; ^3^ Wenzhou Medical University, Department of Radiology, First Affiliated Hospital of Wenzhou Medical University, Wenzhou, Zhejiang, China

**Keywords:** leiomyosarcoma, diagnosis, immunohistochemistry, computed tomography, smooth muscles

## Abstract

Leiomyosarcoma (LMS) is an uncommon and aggressive form of cancer that originates in the smooth muscles. It possesses the capacity for rapid growth and often manifests with general, nonspecific symptoms arising from the displacement of nearby structures rather than direct invasion. In this particular instance, an 81-year-old woman presented with right lower abdominal pain, leading to the discovery of a mass adjacent to the right external iliac artery. In this case, the patient was misdiagnosed initially because of her nonspecific and poorly distinguished clinical symptoms. The laboratory results were within normal ranges. A well-defined tumor was detected through laparoscopic operation and subsequently surgically excised. The histopathological analysis of the tumor revealed the presence of malignant spindle cells, nuclear pleomorphism, and tumor giant cells. Immunohistochemistry tests indicated positive results for CD34 and Desmin, while CD117 and DOG1 showed adverse effects. It is worth noting that LMS of the right external iliac artery is an infrequent occurrence, potentially resulting in delayed diagnosis and misidentification. To enhance our comprehension of this uncommon cancer, more cases with detailed information are essential.

## 1 Introduction

Leiomyosarcoma (LMS) is a sarcoma originating from smooth muscle and can be categorized into two distinct types. The first is the cutaneous type, arising from the arrector pili muscles associated with hair follicles. The second is the subcutaneous type, derived from vascular smooth muscle (1). This malignancy is rare, accounting for only 1% to 4% of all sarcomas. Furthermore, it is recognized that soft tissue sarcomas originating in the extremities tend to exhibit better survival rates compared to those arising in the retroperitoneum (2). LMS typically develops within various anatomical locations, including the alimentary tract, retroperitoneum, genitourinary tract, and soft tissues (3). Vascular leiomyosarcomas are a distinct subset, originating from the muscular walls of major blood vessels, with the inferior vena cava accounting for up to 75% of cases (4). Given the rarity of this malignancy, previous studies have predominantly focused on case reports. Comprehensive assessments, including radiological, biochemical, histomorphological, and immunohistochemical evaluations, are imperative for achieving an accurate diagnosis of leiomyosarcoma. It is within this context that we report this particular case to describe the diagnostic process and share this clinical experience with other health professionals. We hope our report raises awareness among readers and facilitates future diagnoses of similar cases.

## 2 Patient & method

### 2.1 Case report

An 81-year-old female with high blood pressure and diabetes for more than two years. The patient presented with right lower abdominal pain for more than two years. Then, the patient came to our hospital for treatment. A CT examination of the abdomen showed “para-iliac vascular nodules in the right lower abdomen, and an enhanced examination (about 26x19mm) was recommended for the pelvic cavity. Enhanced CT later showed right pelvic iliac vascular nodules; malignancy was considered. Upon admission, the patient’s physical examination revealed no notable findings except for a sizable, firm, and non-painful mass located deep within the hypogastrium. The mass was primarily situated on the right side, positioned above the right psoas muscle, and its lower extent remained inaccessible during the examination.

**Figure 1:**
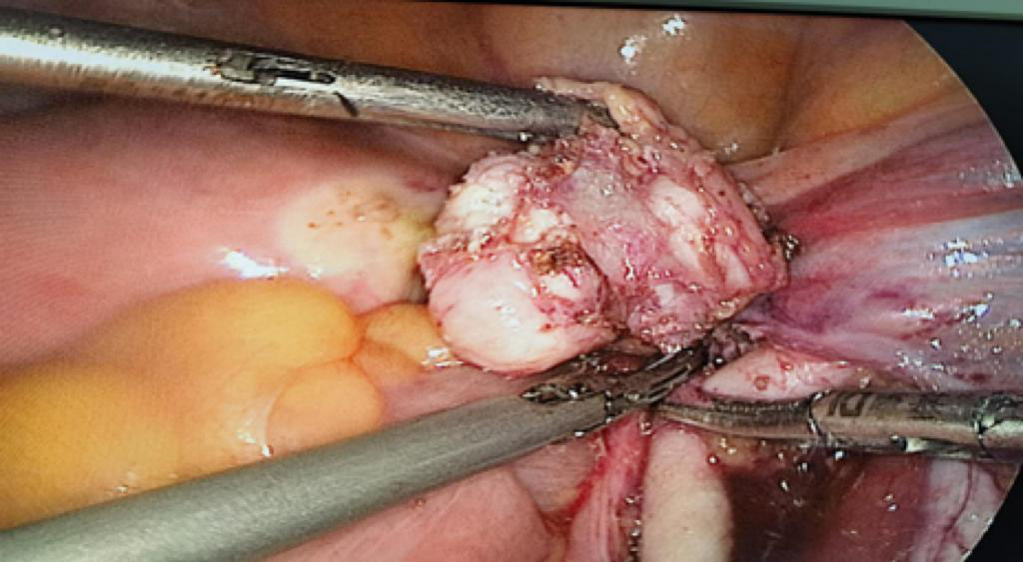
Operation photo showing the mass surrounding the external iliac artery.

Laparoscopic surgery operation report: During the procedure, a mass measuring approximately 3x2 cm in size was identified between the right external iliac artery and the right side of the vein. It became clearly visible following the separation of the external iliac vein. The mass surrounding the right external artery was meticulously excised, as depicted in **Figure 1**. Pathological examination and immunohistochemistry confirmed that the mass was a well-differentiated LMS originating from the external iliac artery. She received radiotherapy after the operation. No local recurrence or distant metastasis during a 6-month follow-up.

### 2.2 Diagnosis procedures and result

#### 2.2.1 Computed tomography

**Figure 2:**
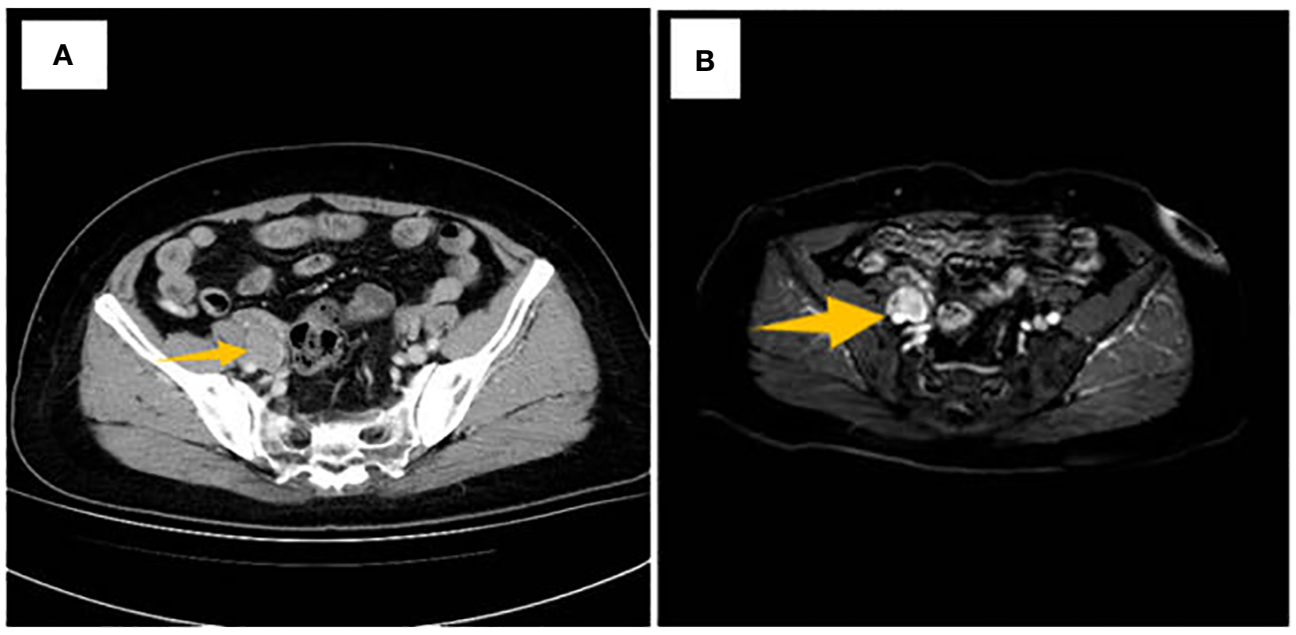
**(A)** CT scan image, the arrow shows the right pelvic iliac vascular nodule, **(B)** MRI imaging showing a lobulated mass presented in the iliac artery.

In **Figure 2A**, it can be noted that on the right side of the pelvis, a group-like shadow next to the local iliac vessels could be seen with a range of about 29×19mm, and the local boundary with the calcified nodules on the right side of the pelvis is unclear. A clear wall surrounds the corresponding area of the iliac arteries. Result diagnosis: On the right side of the pelvic cavity adjacent to the iliac vessel nodules, malignancy should be a primary consideration.

#### 2.2.2 Magnetic resonance imaging

As illustrated in **Figure 2B**, a nodular abnormal signal is evident, featuring a low signal on T1-weighted imaging (T1WI) and a low signal on T2-weighted imaging (T2WI). Following contrast enhancement, mild enhancement is observed, with dimensions measuring approximately 26x18 mm. Furthermore, there is a nodular low signal on T1WI, a slightly elevated signal on T2WI, and a streak-like high signal in the right adnexal region of the right pelvic area. Signal: high signal on DWI, noticeable enhancement after enhancement, size about 2621 mm; the lesion surrounds part of the iliac vessels; several nodular low signals on T2WI in the uterine wall, partially protruding into the uterine cavity; uneven enhancement after enhancement; The largest diameter is about 14 x 12 mm. MRI revealed a well-circumscribed, lobulated, enhancing mass.

#### 2.2.3 Pathological findings

**Figure 3:**
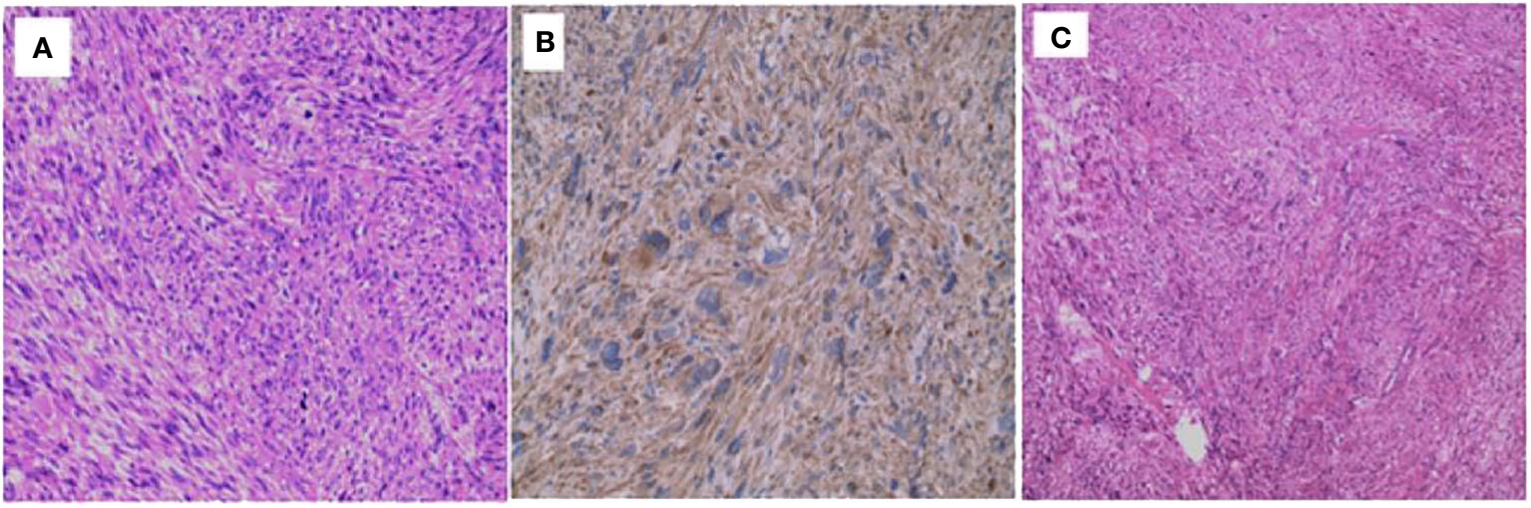
Microscopic Observations **(A, B)** Microscopic analysis revealing nuclear pleomorphism, the presence of giant cells, and the arrangement of spindled neoplastic cells in interlacing bundles. **(C)** Microscopic examination of the tumor mass displaying regions of necrosis and fibrosis within the tissue.

On microscopic examination:

Upon microscopic examination, as depicted in **Figures 3A, B**, the mass displayed a thin capsule. It consisted of spindled neoplastic cells organized in interlacing bundles and fascicles of varying sizes. These individual cells exhibited moderately eosinophilic cytoplasm and cigar-shaped nuclei with dispersed chromatin. Notably, at the tumor’s periphery, a section of the adrenal cortex with a normal appearance was identified, suggesting the tumor’s origin. The tumor was marked by nuclear pleomorphism, the presence of giant cells, and the occurrence of 10 to 12 abnormal mitotic figures per 10 high-power fields. The stroma contained numerous congested blood vessels.

Additionally, it is worth mentioning that the mass displayed significant cytologic atypia, featuring closely packed interlacing bundles of multinucleated, giant spindle cells with elongated, cigar-shaped hyperchromatic nuclei embedded within abundant granular, eosinophilic cytoplasm, as illustrated in **Figure 3C**. Atypical mitotic figures (5 mitoses/ten high-power fields) were also observed. Furthermore, areas of necrosis and fibrosis were evident within the tissue. These findings align with a diagnosis of high-grade soft tissue sarcoma, specifically an arterial leiomyosarcoma.

#### 2.2.4 Immunohistochemical findings

The tumor cells expressed CD34, Desmin, HE (cut), Ki67, S-100, SDHB, and SMA, with no corresponding expression detected in the normal smooth muscle surrounding arterioles in the adjacent area, as indicated in **Table 1**.

**Table 1:** Immunohistochemical findings demonstrating positive Immunostaining Exclusive to Tumor Cells.

primary antibodies	Positivity
**CD34**	**+++**
**Desmin**	**++**
**Ki67**	**++**
**SMA**	**++**
**SDHB**	**++**
**S-100**	**++**

## 3 Discussion

Leiomyosarcomas may arise at any age, although they are commonly diagnosed in individuals over 50 (5). Our patient was an adult female, consistent with previous case reports indicating that soft tissue leiomyosarcoma primarily affects adults (6).

The clinical features of LMS are nonspecific. It primarily depends on the growth pattern, size, and site. Several reports have shown that patients often experience referred pain due to metastatic disease (7). In this case, there was no metastasis; the patient presented a tumor with localized pain. The available data indicates that arterial lesions are predominantly located within the pulmonary artery, with fewer occurrences observed in larger systemic arteries, making reported cases of arterial involvement relatively rare (8). The earliest documented case of leiomyosarcoma in a significant artery dates back to 1909, when Auffermann reported such an occurrence. Subsequently, only a limited number of cases have been reported, including two in the aorta and nine in peripheral arteries (9). Among these reported cases, two were explicitly noted in the common iliac artery, with only one occurring intraluminally. Some studies have described Leriche syndrome as caused by primary intraluminal leiomyosarcoma of the right common iliac artery (10). Our patient represents a unique case, as her tumor is not intraluminal.

Preoperative laboratory results were within normal ranges, and thus, the diagnosis can initially be neglected. In the diagnosis of leiomyosarcomas, advanced imaging techniques such as Computed Tomography (CT) and Magnetic Resonance Imaging (MRI) play a pivotal role in elucidating the characteristics and patterns of neoplastic spread (11). The preoperative CT and MRI, in our case, also played an essential role in showing the mass in the right iliac artery. Notably, microscopic diagnosis of leiomyosarcoma has presented challenges in many cases, although certain histologic features, including cellularity, necrosis, and mitotic activity, have generally served as reliable criteria for identifying malignancy (1). Histopathological characteristics of leiomyosarcoma resemble those found in leiomyosarcomas at other sites, including features such as necrosis, cellular atypia, and mitotic activity (12). While we report these histopathological findings in our case, it is essential to note that accurately diagnosing and predicting the prognosis of leiomyosarcoma through histopathological examination can be challenging.

Consequently, many researchers have explored various diagnostic and prognostic biochemical and molecular markers. Cyclin-dependent kinases (CDKs) are among the markers reported to be present across multiple sarcoma types, including leiomyosarcoma (13). In our study, we observed the presence of CD34 around arterioles adjacent to the tumor site. Additionally, our case tested positive immunohistochemically for Desmin, HE (cut), Ki67, S-100, SDHB, and SMA, commonly used markers. All reported cases were incidentally diagnosed intraoperatively, postoperatively, or during autopsy. Surgical procedures are detailed in our case study, helping specialists confirm the diagnosis of this rare tumor. Ultrasonographic examination of the affected vessels and pelvic organs may be beneficial in cases involving primary pelvic masses.

Further investigation, extended follow-up, or autopsy can provide definitive diagnoses. Microscopically, most reported cases of tumors in large arteries have been identified as leiomyosarcomas, originating from or associated with the vessel wall, as confirmed in our study (14). Spindle cells are typically a hallmark of such tumors, often accompanied by subintimal fibrous changes. The number of mitotic images per ten high-power fields serves as a prognostic indicator, addressing the challenges researchers face in this field. While histological characteristics such as atypia, cellularity, and necrosis are associated with malignancy to some extent, the mitotic count per high-power field (HPF) appears to be the most reliable indicator of malignancy (15).

## 4 Conclusion

Our case study underscores the rarity and aggressiveness of arterial leiomyosarcomas. Timely and precise diagnosis plays a crucial role in enhancing the prognosis of these tumors. A comprehensive medical evaluation encompassing radiological, biochemical, histomorphological, and immunohistochemical analyses is imperative for the accurate identification of LMS. This motivation prompted our in-depth examination of this case.

## Data availability statement

The datasets presented in this study can be found in online repositories. The names of the repository/repositories and accession number(s) can be found in the article/supplementary material.

## Ethics statement

The studies involving humans were approved by Gynecology department of the First Affiliated Hospital of Wenzhou Medical University. The studies were conducted in accordance with the local legislation and institutional requirements. The participants provided their written informed consent to participate in this study. Written informed consent was obtained from the individual(s) for the publication of any potentially identifiable images or data included in this article.

## Author contributions

MO: Conceptualization, Formal Analysis, Methodology, Writing – original draft. AR: Conceptualization, Formal Analysis, Methodology, Writing – original draft, Investigation, Writing – review & editing. WY: Formal Analysis, Investigation, Writing – original draft. YB: Data curation, Methodology, Software, Writing – original draft. QZ: Conceptualization, Methodology, Validation, Writing – original draft. XW: Data curation, Investigation, Writing – original draft. GC: Funding acquisition, Investigation, Supervision, Writing – original draft. XY: Funding acquisition, Investigation, Supervision, Writing – original draft.
